# Comparison of 3D facial photographs and clinical documentation in patients with craniofacial morphea

**DOI:** 10.1002/ski2.438

**Published:** 2024-08-10

**Authors:** Tyler T. Nguyen, Stephanie M. Cohen, Katharina S. Shaw, Fatma Dedeoglu, Ruth Ann Vleugels, Ingrid M. Ganske

**Affiliations:** ^1^ Department of Plastic and Oral Surgery Boston Children's Hospital and Harvard Medical School Boston Massachusetts USA; ^2^ Department of Surgery Beth Israel Deaconess Medical Center Boston Massachusetts USA; ^3^ Section of Dermatology Children's Hospital of Philadelphia Philadelphia Pennsylvania USA; ^4^ Rheumatology Program Division of Immunology Boston Children's Hospital and Department of Pediatrics Harvard Medical School Boston Massachusetts USA; ^5^ Dermatology Program Division of Immunology Boston Children's Hospital and Harvard Medical School Boston Massachusetts USA; ^6^ Department of Dermatology Brigham and Women's Hospital Boston Massachusetts USA

## Abstract

Diagnosis of craniofacial morphea (CM) relies upon clinical examination of progressive craniofacial changes. We assess the utility of 3D stereophotogrammetry in documenting asymmetry of the face compared to clinical notetaking. This retrospective study of 3D images and clinical documentation included 32 patients (mean age 15.7 years) with CM. A panel of specialists identified additional areas of asymmetry (those highlighted in 3D photographs that were not noted in clinical documentation) and categorised them as *likely*, *ambiguously* or *unlikely* related to CM. 28 patients (87.5%) had asymmetries noted on 3D photos that were not documented in clinical notes. In 46.4% of them, additional areas were deemed consistent with CM. In the remaining patients, additional asymmetries were ambiguous (42.9%) or not thought to be related to CM (10.7%). Our results suggest that adjunctive use of 3D stereophotogrammetry enhances the documentation of CM at discrete clinical time points and therefore could be a better comparative reference during later re‐examination.

## Graphical abstract

Craniofacial morphea (CM) is a rare autoimmune disease characterised by progressive atrophy of the skin and soft tissue of the face, resulting in facial asymmetry. With the involvement of varying practices in the care of patients with CM, consistent documentation of disease location and severity lack standardized evaluation. In comparing clinical documentation to mirror‐imaged 3‐dimensional (3D) photos, our results suggest that the adjunctive use of 3D stereophotogrammetry may enhance clinical documentation of CM disease.

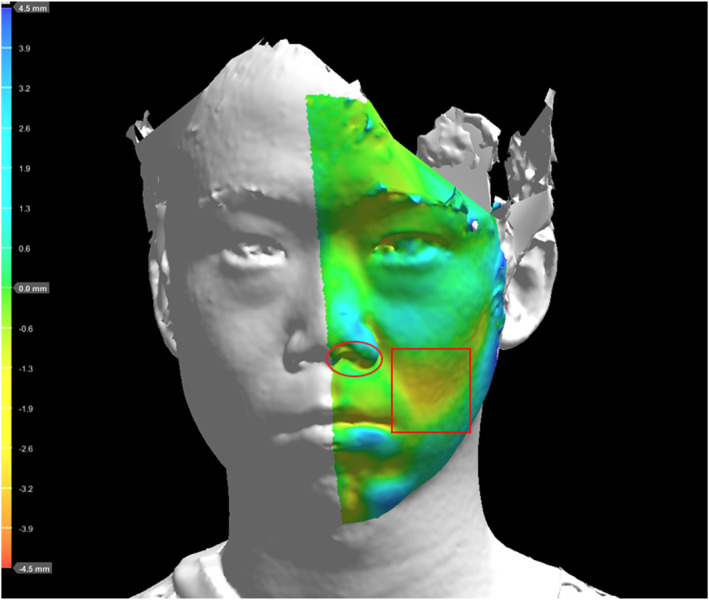

## 1 INTRODUCTION

Craniofacial morphea (CM; encompassing frontoparietal linear morphea, en coup de sabre, progressive hemifacial atrophy and Parry–Romberg Syndrome) is a rare autoimmune disorder characterised by progressive atrophy of skin and underlying structures. The diagnosis is based on signs of active sclerosis (erythema, pigmentary changes and induration), resulting in structural damage including alopecia and volumetric atrophy/asymmetry. Physical examination may be paired with validated clinical tools for detecting morphea disease activity and damage including the localized scleroderma assessment tool (LoSCAT),^1^ localised scleroderma skin damage index (LoSDI)^2^ or morphea activity measure.^3^ Because facial damage from CM accrues gradually, these metrics can be limited in ability to assess and record subtle changes over time.^4^ Additionally, various non‐craniofacial clinicians who treat this condition (rheumatology, dermatology, neurology and primary care) may be less versed in assessing facial asymmetry.

Given the multidisciplinary nature of CM, physicians often evaluate patients at different time points and describe disease locations with varying degrees of detail and precision. As such, there is a critical need to quantify and standardise reporting of the soft tissue atrophy of CM. While magnetic resonance imaging (MRI) has been piloted in CM, serial imaging with this modality is often impractical and potentially unsafe, particularly in young patients who require sedation. As such, non‐invasive and radiation‐free techniques (i.e., 3D stereophotogrammetry) are increasingly considered to be more favourable due to a significantly rapid photo acquisition time.^5^, ^6^ Thus, we aimed to understand how well 3D stereophotogrammetry could document asymmetry of the face compared to the current clinical note‐taking strategies.

## 2 REPORT

We performed a retrospective review of patients with CM treated at Boston Children's Hospital from April 2019 to April 2022. In the multidisciplinary care of CM patients evaluated in our combined paediatric Rheumatology/Dermatology clinic, 2D photos and 3D stereophotogrammetry were obtained from all patients. Inclusion criteria: patients with CM of all ages. Exclusion criteria: patients with a history of reconstructive procedures performed prior to the study, uncertain diagnosis of CM or a concurrent diagnosis affecting facial symmetry (e.g., trauma). The Institutional Review Board at Boston Children's Hospital approved all research activities and informed consent for participation and for the use of facial photographs was obtained from all participants in this study.

All clinical assessments recorded in the electronic medical record were analysed and the location of facial asymmetry and disease status were noted. Atrophy and/or skin lesions other than on the face were not considered.

Patient photographs were captured in the plastic surgery clinic by trained clinical staff during each visit to our institution. Static 2D photos captured the entire head and neck at six different angles to simulate the physician's point of view during examination. 3D facial photographs were taken using VECTRA M5 3D Imaging System (Canfield Scientific). VECTRA software was used for post‐processing analysis to mirror image and identify asymmetry in heat maps of each photo.^7^

**FIGURE 1:**
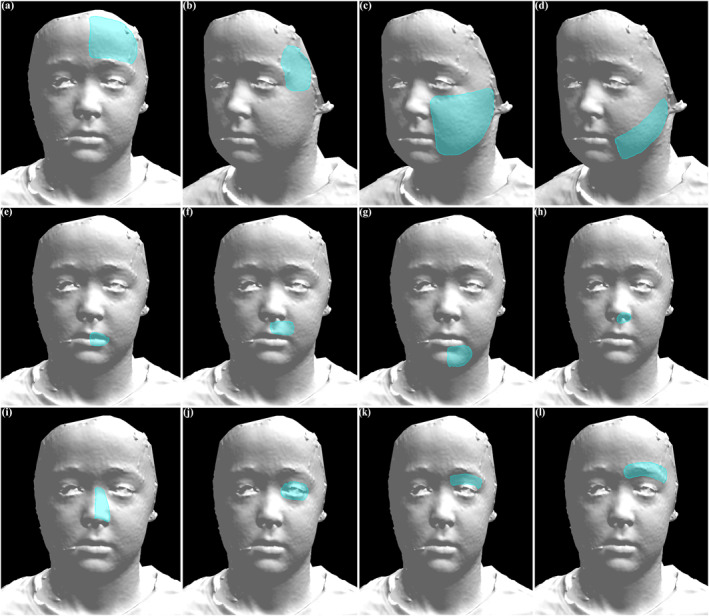
Anatomic regions of 3‐dimensional facial photographs are shown. Unilateral regions are shown but can be applied to the contralateral side depending on the location of patient disease. (a) Forehead. (b) Temple. (c) Cheek. (d) Jaw/Jawline. (e) Lip—Vermilion. (f) Lip—Cutaneous. (g) Chin. (h) Nose—Nostril/Alar base. (i) Nose—Nasal Side Wall. (j) Eye—Globe. (k) Eye—Eyelid. (l) Brow.

Mirror‐imaged photos were reviewed and categorised based on laterality (left vs. right) and facial region (Figure 1). Clinical documentation of facial atrophy was compared to flip‐registered 3D photos and static 2D photos. Asymmetrical facial regions identified through visual heat mapping in 3D facial photographs that were not mentioned in clinical documentation were deemed ‘additional areas of asymmetry’.

**FIGURE 2:**
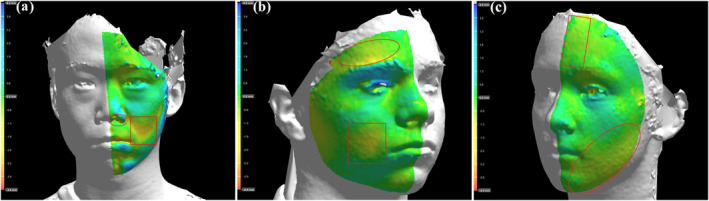
All 3‐dimensional photos are shown on a 4.5 mm scale. Areas in green indicate little to no facial asymmetry to the contralateral side. Areas in yellow, orange and red indicate increasing difference in facial volume compared to the contralateral side. Squared regions are clinically documented disease. Circled regions are identified additional areas of asymmetry. (a) Example of additional area likely consistent with the disease. (b) Example of additional area ambiguously related to the disease. (c) Example of additional area unlikely consistent with the disease.

A panel review of four CM experts and two non‐experts in plastic surgery, dermatology and rheumatology assessed whether additional areas of asymmetry were likely to reflect CM. The panel met together and reviewed 2D and 3D photographs. The number and location of ‘additional areas of asymmetry’ were noted. When all panellists were in agreement, the area was designated as ‘likely consistent’ with CM; if panellists were unsure or there was a discrepancy in opinion, it was designated as ‘ambiguously consistent’; when all were in agreement that an area was not related to CM, it was designated as ‘likely inconsistent’. (Figure 2). All patients with additional areas of asymmetry were flagged for close examination at their next clinical follow‐up. Due to the retrospective nature of the study, not all patients were undergoing active treatment to facilitate reassessment.

The study group consisted of the remaining 32 patients (22 female) with an average age of 15.7 years (range 5.3–40.5) at first intake. Within this cohort, 28 (87.5%) patients had ‘additional areas of asymmetry’. For the majority of patients (*n* = 13), there was one additional asymmetrical facial region that had not been noted in clinical documentation. Patients with two (*n* = 11) or three additional facial locations (*n* = 4) were also noted.

Upon panel review, 13 out of the 28 patients (46.4%) with ‘additional areas of asymmetry’ were identified as having asymmetry *likely consistent* with CM. Additional areas of asymmetry for 12 patients (42.9%) were flagged as *ambiguous* based on the photographs alone and require future follow‐up to further assess the likely aetiology of the facial asymmetry noted. Finally, three patients (10.7%) had additional areas of asymmetry *likely inconsistent* with CM.

## 3 DISCUSSION

Our study examines the utility of routine 3D stereophotogrammetry to enhance documentation of facial asymmetry in CM. Visualising volumetric differences through the mirror imaging process thoroughly demarcates anomalous facial geography for immediate and future reference. In nearly half of the patients with CM, we identified additional regions of CM‐related facial atrophy using 3D stereophotogrammetry that were clinically documented, indicating that the imaging may give more information and/or better record what is evident than simply describing findings in clinical notes.

Early signs of CM are often subtle, and 3D stereophotogrammetry depicts the face volumetrically in a manner that may reveal areas of asymmetry missed by clinical examination alone. Additionally, determining if the disease is progressing affects management decisions; therefore, being able to assess minor changes between clinical time points is paramount. The benefits of 3D stereophotogrammetry for assessing facial asymmetry and atrophy are widely reported in plastic surgery. However, there are few studies on its application in dermatological diseases such as CM. We have found 3D stereophotogrammetry useful for evaluating CM status and progression,^5^, ^8^ and others have examined its use for reconstructive outcomes assessment.^9^, ^10^ This study demonstrates its value in serving as a better record of physical examination than clinical note writing. As the technology becomes more financially accessible and protocols are standardized for rapid assessment of soft tissue atrophy, 3D stereophotogrammetry may also prove a highly valuable tool for early detection of facial asymmetry and improved patient outcomes, as well as inter‐institutional collaboration and research.

The differences between 3D stereophotogrammetry findings and clinical documentation do not exclusively point to deficiency in physicians' clinical detection of disease. Rather, these differences likely reflect a combination of factors including clinician recall, the areas of primary focus by the patient/family, clinician focus on identifying active disease instead of areas of expected atrophy and inconsistent details in reporting of facial regions between providers. While there are objective instruments for morphea in general,^1^, ^2^, ^3^ these are insufficient to categorise the CM subtype, as they do not capture the functional and aesthetic impact on the face. Such scales for general skin and soft tissue involvement could be enhanced with the addition of 3D imaging to capture damage to specific facial features.

Clinical interpretation of 3D imaging is important. Varying degrees of facial asymmetry are commonly encountered in the absence of morphea; and it is worth noting that 42.9% of patients had areas of additional minor facial asymmetry noted in their 3D images requiring correlation with a repeat physical exam to determine if they were the result of CM. Being able to review 3D images in real time during the clinical exam gives providers the opportunity to assess areas of ambiguity and flag them for appropriate monitoring on subsequent exams. Additionally, having a baseline set of 3D images on patients without CM would help provide further context for interpreting subtle positive findings.

An ability to comprehensively and objectively characterise areas of facial asymmetry in CM makes this imaging modality—in conjunction with existing 2D imaging and clinical documentation—a promising tool for improving the quality of care for patients with this orphan disease. Further studies are necessary to determine the efficacy, cost implications and best practices for incorporating 3D stereophotogrammetry into the evaluation and decision‐making for patients with CM.

## CONFLICT OF INTEREST STATEMENT

None to declare.

## AUTHOR CONTRIBUTIONS

**Tyler T. Nguyen**: Conceptualization (equal); data curation (lead); formal analysis (lead); investigation (equal); methodology (equal); project administration (lead); software (lead); writing – original draft (lead); writing – review & editing (equal). **Stephanie M. Cohen**: Conceptualization (equal); data curation (supporting); formal analysis (equal); investigation (supporting); methodology (supporting); validation (supporting); writing – review & editing (supporting). **Katharina S. Shaw**: Conceptualization (supporting); data curation (supporting); formal analysis (equal); investigation (equal); methodology (equal); validation (equal); writing – original draft (supporting); writing – review & editing (supporting). **Fatma Dedeoglu**: Conceptualization (supporting); data curation (supporting); formal analysis (equal); investigation (equal); methodology (equal); supervision (supporting); validation (equal); writing – original draft (supporting); writing – review & editing (supporting). **Ruth Ann Vleugels**: Conceptualization (equal); data curation (supporting); formal analysis (equal); investigation (equal); methodology (equal); supervision (equal); validation (equal); writing – original draft (supporting); writing – review & editing (equal). **Ingrid M. Ganske**: Conceptualization (lead); data curation (equal); formal analysis (equal); investigation (equal); methodology (equal); project administration (equal); supervision (lead); writing – original draft (equal); writing – review & editing (lead).

## ETHICS STATEMENT

The Institutional Review Board at Boston Children's Hospital approved this research study (IRB‐P00029401). All research activities adhere to the tenets of the Declaration of Helsinki and all informed patient consent has been obtained.

## PATIENT CONSENT

Written patient consent for publication was obtained.

## Data Availability

The data from this study and its accompanying photos are available on reasonable request from the corresponding author. The data are not publicly available due to privacy concerns with the full‐facial photographs obtained.

## References

[1] Kelsey CE , Torok KS . The localized scleroderma cutaneous assessment tool: responsiveness to change in a pediatric clinical population. J Am Acad Dermatol. 2013;69(2):214–220. 10.1016/j.jaad.2013.02.007 23562760 PMC3720681

[2] Arkachaisri T , Vilaiyuk S , Torok KS , Medsger TA . Development and initial validation of the localized scleroderma skin damage index and physician global assessment of disease damage: a proof‐of‐concept study. Rheumatol Oxf Engl. 2010;49(2):373–381. 10.1093/rheumatology/kep361 PMC3498950 20008472

[3] García‐Romero MT , Tollefson M , Pope E , Brandling‐Bennett HA , Paller AS , Keimig E , et al. Development and validation of the morphea activity measure in patients with pediatric morphea. JAMA Dermatol. 2023;159(3):299–307. 10.1001/jamadermatol.2022.6365 36753150 PMC9909574

[4] Agazzi A , Fadanelli G , Vittadello F , Zulian F , Martini G . Reliability of LoSCAT score for activity and tissue damage assessment in a large cohort of patients with Juvenile Localized Scleroderma. Pediatr Rheumatol Online J. 2018;16(1):37. 10.1186/s12969-018-0254-9 29914516 PMC6006585

[5] Ganske IM , Cappitelli AT , Langa OC , Min M , Torok KS , Dedeoglu F , et al. Pilot use of 3‐dimensional photography to aid clinical decision‐making in craniofacial morphea. JAAD Case Rep. 2022;25:100–103. 10.1016/j.jdcr.2022.05.038 35794965 PMC9251559

[6] Vallen H , Xi T , Nienhuijs M , Borstlap W , Loonen T , Hoogendoorn B , et al. Three‐dimensional stereophotogrammetry measurement of facial asymmetry in patients with congenital muscular torticollis: a non‐invasive method. Int J Oral Maxillofac Surg. 2021;50(6):835–842. 10.1016/j.ijom.2020.09.011 33069517

[7] Yu Z , Mu X , Feng S , Han J , Chang T . Flip‐registration procedure of three‐dimensional laser surface scanning images on quantitative evaluation of facial asymmetries. J Craniofac Surg. 2009;20(1):157–160. 10.1097/scs.0b013e318191ce88 19165015

[8] Shaw KS , Nguyen TT , Rajeh A , Cohen SM , Semenov YR , Reusch DB , et al. Use of 3‐dimensional stereophotogrammetry to detect disease progression in craniofacial morphea. JAMA Dermatol. 2023:e233649. 10.1001/jamadermatol.2023.3649 PMC10568443 37819665

[9] Pucciarelli V , Baserga C , Codari M , Beltramini GA , Sforza C , Giannì AB . Three‐dimensional stereophotogrammetric evaluation of the efficacy of autologous fat grafting in the treatment of parry‐romberg Syndrome. J Craniofac Surg. 2018;29(8):2124–2127. 10.1097/scs.0000000000004664 29894458

[10] Ter Horst R , Maal TJJ , de Koning MJJ , Mertens JS , Schatorjé EJH , Hoppenreijs EP , et al. 3D stereophotogrammetry in children and adolescents with Scleroderma En Coup De Sabre/Parry‐Romberg Syndrome: description of a novel method for monitoring disease progression. Skin Health Dis. 2022;2(3):e132. 10.1002/ski2.132 36092259 PMC9435452

